# Evaluation of the Efficacy of Nasal Sedation Midazolam Compared with Dexmedetomidine in the Management of Uncooperative Children with Down Syndrome during Dental Treatment

**DOI:** 10.1155/2022/7344928

**Published:** 2022-09-16

**Authors:** Mohamad Nabil Hamod, Chaza Kouchaji, Faten Rostom, Hasan Alzoubi, Imad Katbeh, Nikolay Tuturov

**Affiliations:** ^1^Department of Pediatric Dentistry, Faculty of Dentistry, Damascus University, Damascus, Syria; ^2^Department of Anesthesia and Reanimation, Faculty of Medicine, Damascus University, Damascus, Syria; ^3^Department of Pediatric Dentistry and Orthodontics, RUDN University (Peoples' Friendship University of Russia), Moscow, Russia

## Abstract

**Objective:**

This study aimed to compare the intranasal administration of midazolam and dexmedetomidine in uncooperative children with Down syndrome.

**Materials and Methods:**

The sample consisted of 20 children with Down syndrome aged 5 to 11 years who were divided equally into two groups: Group 1 (experimental) nasal dexmedetomidine and Group 2 (control) nasal midazolam. The efficacy of both the drugs was evaluated according to Ohio State University Behavioral Rating Scale (OSUBRS), University of Michigan scale (UMSS), and Houpt general behavior scale.

**Results:**

Both substances have been effective in the management of children with Down syndrome. There were no statistically significant differences for Ohio State University Behavioral Rating Scale (OSUBRS) (*P* value = 0.631), University of Michigan scale (UMSS) (*P* value = 0.739), and Houpt general behavior scale (*P* value = 0.481).

**Conclusion:**

Both midazolam and dexmedetomidine nasal can be used to sedate children with Down syndrome.

## 1. Introduction

Down syndrome is a chromosomal syndrome caused by a change in chromosomes where there is an extra copy of chromosome 21 or part of it, causing a change in the genes [1].

This syndrome is characterized by changes in the structure of the body, and the syndrome is often accompanied by weakness in mental abilities and physical development and distinctive facial features [2]. People with Down syndrome are characterized by a small chin, enlargement, and protrusion of the tongue due to a small oral cavity, and congenital defects in the heart, and the majority of people with Down syndrome have mental retardation ranging from mild (IQ 50–70) to medium (IQ 35–50) [3].

Pharmacological sedation is defined as a technique in which one or more drugs are used to depress the patient's central nervous system, thereby reducing the patient's awareness of his surroundings. The American Academy of Pediatric Dentistry [4] divided the levels of sedation according to the degree of inhibition of the central nervous system into minimal sedation, moderate sedation (conscious sedation), and deep sedation [5].

Nasal sedation is more effective than the oral route and is preferred to be applied especially in young children. The technique of its application is relatively simple and painless and requires less patient cooperation compared to oral sedation, and it has a quick onset of the effect (10 minutes) [6].

Dexmedetomidine was approved by the FDA as a short-term analgesic and sedative in intensive care patients in 1999, and in 2008 the FDA recommended the use of dexmedetomidine as a sedative for both surgical and nonsurgical procedures [7].

Dexmedetomidine is metabolized in the liver, so it is used with caution in patients with liver problems. Most of the drug is disposed of in the urine (95%). Dexmedetomidine does not alter the patient's respiratory capacity and is therefore used safely in patients with a tendency to develop respiratory depression [8]. Dexmedetomidine has a biphasic effect on blood pressure, it causes a decrease in blood pressure at low concentrations of it, and an increase in blood pressure occurs at high concentrations [9].

Dexmedetomidine showed great efficacy as a sedative when used in dental treatments, and it became widely used due to the absence or lack of complications, as dexmedetomidine does not cause respiratory depression compared to benzodiazepines, opioids, and propofol. However, dexmedetomidine was shown to induce less amnesia compared to benzodiazepines [8].

Nasally applied dexmedetomidine produces a good level of sedation with the absence of complications following sedation. In contrast to nasal midazolam, which causes discomfort in children and nasal irritation [10].

## 2. Materials and Methods

The study protocol was approved by the Scientific Research and Postgraduate Board of Damascus University Ethics Committee of Damascus University, Damascus, Syria (IRB no.UDDS-253-23102017/SRC-1900). A detailed information sheet was provided in advance, and parents/guardians were requested to sign an informed consent. The patients and parents were blinded by not being provided any information about the treatment.

**Figure 1:**
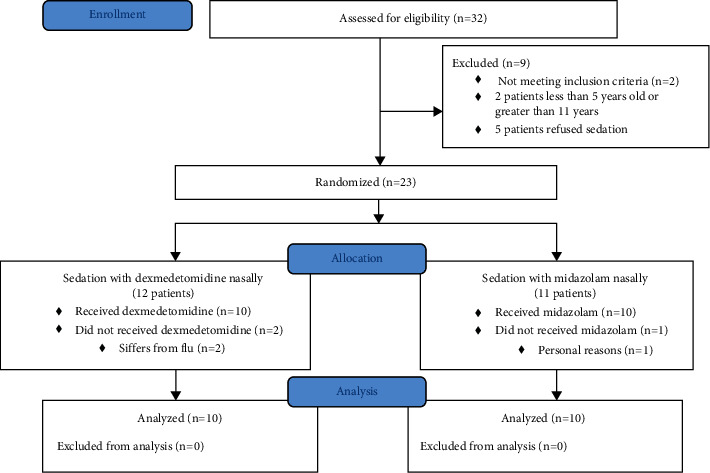
CONSORT flow diagram.

The sample included 20 patients (Figure 1) with Down syndrome who attended the Faculty of Dentistry, Department of Pediatric Dentistry at Damascus University, to compare the efficacy and safety of midazolam and dexmedetomidine in managing patients with Down syndrome and monitoring behavioral change and clinical signs after applying sedative drugs.

The studied sample was randomly distributed at https://www.randomization.com into two groups:

Group *A* (represented the experimental group in which dexmedetomidine was applied nasally).

Group *B* (represented the control group in which midazolam was applied nasally).

a double blinded approach was adopted in this study so that both the patient and the examiner would not know about the applied drug.

General criteria for inclusion in the sample: patient's behavior is within the first degree (absolute negative) or the second degree (negative) of the Frankl scale, the patient should be classified as ASA (I and II), patient's age ranged from 5–11 years and the dental treatment includes a quarter of the jaw (endodontic treatment—conservative treatment—extraction); while patients who received sedative medications within the 48 hours preceding treatment were excluded.

After making sure that the patient fulfilled the inclusion criteria, the written informed consent of the parents was obtained. The form for each child was filled out with his personal information and the child's weight and vital signs were recorded: systolic blood pressure (SBP), diastolic blood pressure (DBP), pulse rate (PR), SpO2, and respiration rate (RR). During the treatment, the patient was monitored according to the guidelines of the American Academy of Pediatric Dentistry, where the aforementioned vital signs were recorded every 5 minutes until the end of the treatment.

**Figure 2:**
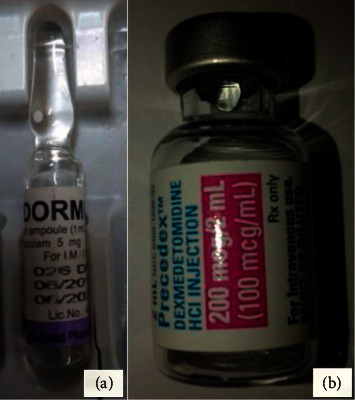
Sedative used drug (a) midazolam and (b) dexmedetomidine.

Dexmedetomidine was used at a dose of 1 mcg/kg (Figure 2), while midazolam was used at a dose of 0.2 mg/kg (Figure 2), where the drug was placed in a syringe and divided into the two nostrils equally. The dose administered to each child and the onset of action (the time required after the drug was administered to make the therapeutic procedures possible) were documented on each patient's form.

After administration of the sedative drug, the following onset signs have waited: drowsiness, slowed eye movement, decreased neuromuscular balance, slurred speech, and sleep. After noticing the signs of sedation, appropriate dental treatment was performed.

During the treatment, it was monitored: vital signs, behavioral response, behavioral response according to the OSUBRS scale (Table 1), level of sedation obtained according to the USMSS scale (Table 2), and reactions and adverse events associated with sedation.

The treatment success rate was estimated by the overall behavior rating using the Houpt General Behavior Scale, where this scale is divided into six scores, starting with score 1 (fail) and ending with score 6 (excellent), where scores 1 and 2 were considered a failure of the sedation process, while the rest of the scores were considered a success (Table 3).

After completing the treatment, the child was moved to a comfortable place until he met the criteria for recovery from the sedation process: the patient was rated 1 or 2 on the modified Vancouver scale (Table 4), some oral questions were answered and vital signs were checked. The child's parents were also contacted on the evening of the treatment day to record any complications if they occur.

## 3. Results

The study sample consisted of 20 children with Down syndrome, their ages ranged between 5 and 11 years, with an average of 7.9 ± 0.9 years. Data were collected and recorded on Microsoft Excel, and data were analyzed using SPSS v.25 (IBM, USA) with a significance level of 0.05.

The Mann–Whitney *U* test was used to study the difference in the scores of OSUBRS, University of Michigan Scale (UMSS), and Houpt General Behavior Scale between the two study groups.

### 3.1. Ohio State University Behavioral Rating Scale (OSUBRS)

In the dexmedetomidine group, 50% of patients had score 1 (calm without movement), 30% score 2 (crying without resistance), and 20% score 3 (movement with resistance without crying) on the Ohio State University Behavioral Rating Scale (OSUBRS), while in Midazolam group, 40% of the score 1, 30% of the score 2, and 30% of the score 3, but there was no statistically significant difference between the two groups (*P* value = 0.631), as seen in Table 5.

### 3.2. Depth of Sedation Scale According to the University of Michigan Scale (UMSS)

In the dexmedetomidine group, 30% of patients had score 1 (slightly sedated) and 70% score 2 (moderately sedated) according to the UMSS, while in the midazolam group, 40% had score 1 and 60% score 2. There was no statistically significant difference between the two groups (*P* value = 0.739), as seen in Table 5.

### 3.3. Houpt General Behavior Scale

In the dexmedetomidine group 20% of patients got score 4 (good), 20% score 5 (very good), and 60% got score 6 (excellent), while in the midazolam group, 30% got score 4 (good), 30% got score 5 (very good), and 40% got score 6 (excellent). However, there was no statistically significant difference between the two groups (*P* value = 0.481), as seen in Table 5.

### 3.4. Vital Signs

There were no statistically significant differences between the two study groups in the mean of systolic blood pressure and pulse rate before and after the treatment (Table 6).

As for the mean of systolic blood pressure during treatment, there were statistically significant differences (*P* value = 0.015), where the values of the systolic blood pressure rate in the dexmedetomidine group were greater than in the midazolam group. As for the mean pulse rate during treatment, there were statistically significant differences (*P* value = 0.011), where the values of the pulse rate in the midazolam group were greater than in the dexmedetomidine group (Table 6).

There were no statistically significant differences between the two study groups in the means of diastolic blood pressure and oxygen saturation before, during, and after work (Table 6).

## 4. Discussion

Sedation is one of how the behavior of uncooperative children and children with special needs can be controlled [11, 12]. The current study was based on comparing the sedation of children with special needs using nasal midazolam and comparing it with dexmedetomidine.

In this study, 20 children with Down's disease were sedated and they were divided into the two study groups equally to show that there is a difference in the level of sedation and the children's behavior during sedation or not.

Children with Down syndrome who are uncooperative within the first and second degree according to the Frankl scale and who show behavior that refuses treatment, as there is difficulty in conducting treatment using the psychological behavioral management methods, and therefore they need treatment under pharmacological sedation, were selected.

This study was characterized by accurate and up-to-date use of appropriate behavioral and sedation measures to assess children's behavior before, during, and after treatment. The Ohio State University Behavioral Rating Scale was used to assess the behavioral response of each child during treatment and to study the effectiveness of the sedation methods as it is characterized by ease, clarity, and reliability [13].

The University of Michigan sedation scale was also used to assess the depth of sedation as it is a simple scale that facilitates frequent assessment and documentation of the depth of sedation in children. The Houpt scale was also used to determine the child's behavior after sedation, as it is a specific scale and to give us a final result of the effectiveness of sedation [14].

70% of the dexmedetomidine group scored 2 on the University of Michigan scale (moderately sedated), while in the midazolam group, 60% scored a grade of 2, with no significant difference in depth of sedation among the study population.

This study is consistent with the study by Miller et al. which found that nasal application of dexmedetomidine induced a good sedation level in Down patients undergoing ECG [15].

This study also agrees with the study of Mahdavi et al. which evaluated the nasal preparation of the drug in noncooperative children who received midazolam at a dose of 0.5 mg/kg or dexmedetomidine at a dose of 1 mcg/kg, where there were no statistical differences in the efficacy of the nasal drug preparation between dexmedetomidine and midazolam [16].

In addition, this study agreed with the study of Akin et al. who made a drug preparation before general anesthesia, where they used doses similar to those used in this study, and they found that the sedative efficacy of both dexmedetomidine and nasal midazolam was equal in reducing anxiety when children were separated from their parents [17].

These results differ from the study of Sheta et al. which found that the level of sedation was higher in the dexmedetomidine group, and this can be attributed to the different age groups studied and the characteristics of this group [10].

It also differs from Suredar et al. study, which found that the highest success rate for behavior management was in the dexmedetomidine group and this difference is because Natarajan Suredar's study compared two doses of dexmedetomidine, and the success rate of behavior management increased with the increasing dose [11].

## 5. Conclusion

Among the limitations of this study, both nasal midazolam and dexmedetomidine can be considered effective in the management of children with Down syndrome.

### 5.1. Limitation

A limitation of this study was the small sample size where subjects were Down syndrome children and the inability to blind the responsible for drug administration.

## Figures and Tables

**Table 1:** Ohio State University Behavioral Rating Scale (OSUBRS).

Score 1	Calm and no movement.
Score 2	Crying without resistance.
Score 3	Movement with resistance without crying.
Score 4	Movement with resistance with crying

**Table 2:** Depth of the sedation scale according to the University of Michigan scale (UMSS).

Score 0	Completely awake.
Score 1	Slightly sedated, tired, relaxed, and responds to speech or voice prompts.
Score 2	Moderately sedated, drowsy, asleep, and responds easily to light tactile stimulations or verbal commands
Score 3	Deeply sedated, deeply asleep, and responds only to strong physical stimulations.
Score 4	Unresponsive.

**Table 3:** Houpt general behavior scale.

Scores	Type of behavior	Behavioral assessment	Result of the sedation system
1	Failure	It was not possible to do the treatment at all.	Failure
2	Bad	Treatment was discontinued and only partial treatment was done.	
3	Moderate	Intermittent treatment but completed treatment.	Success
4	Good	Crying or moderate movement did not affect the treatment.	
5	Very good	Some crying and limited movement.	
6	Excellent	No crying or movement.	

**Table 4:** Modified Vancouver scale to assess recovery from sedation.

Scores	Behavioral assessment
1	Completely awake.
2	Eyes open: the patient responds to verbal questions.
3	Eyes open: the patient does not respond to verbal questions.
4	Eyes closed: the patient does not respond to verbal questions.
5	Eyes closed: the patient is aroused by slight agitation.
6	Eyes closed: the patient is not aroused by slight agitation.

**Table 5:** Results of the studied scales.

Scales	Scores	Dexmedetomidine		Midazolam		*P* value
OSUBRS	Score 1	**5**	**50%**	**4**	**40%**	0.631
	Score 2	**3**	**30%**	**3**	**30%**	
	Score 3	**2**	**20%**	**3**	**30%**	
	Score 4	**0**	**0%**	**0**	**0%**	
UMSS	Score 0	**0**	**0%**	**0**	**0%**	0.739
	Score 1	**3**	**30%**	**4**	**40%**	
	Score 2	**7**	**70%**	**6**	**60%**	
	Score 3	**0**	**0%**	**0**	**0%**	
	Score 4	**0**	**0%**	**0**	**0%**	
Houpt	Score 1	**0**	**0%**	**0**	**0%**	0.481
	Score 2	**0**	**0%**	**0**	**0%**	
	Score 3	**0**	**0%**	**0**	**0%**	
	Score 4	**2**	**20%**	**3**	**30%**	
	Score 5	**2**	**20%**	**3**	**30%**	
	Score 6	**6**	**60%**	**4**	**40%**	

**Table 6:** Results of vital sign analyzes.

	Stages	Sedation methods	Mean	SD	*t*-value	*P* value
SBP	Before treatment	Dexmedetomidine	102.50	3.25	−1.672	0.31
		Midazolam	102.83	2.92		
	During treatment	Dexmedetomidine	100.36	3.91	3.272	0.015
		Midazolam	95.55	3.18		
	After treatment	Dexmedetomidine	101.95	2.87	−2.021	0.23
		Midazolam	103.21	2.66		
DBP	Before treatment	Dexmedetomidine	68.97	2.96	−2.034	0.83
		Midazolam	69.42	3.22		
	During treatment	Dexmedetomidine	67.12	3.72	1.462	0.17
		Midazolam	65.89	4.05		
	After treatment	Dexmedetomidine	69.28	3.53	0.945	0.42
		Midazolam	68.45	3.55		
PR	Before treatment	Dexmedetomidine	106.5	9.7	2.580	0.266
		Midazolam	105.8	8.9		
	During treatment	Dexmedetomidine	99.7	6.5	−1.044	0.011
		Midazolam	106.4	8.2		
	After treatment	Dexmedetomidine	105.5	7.3	−1.053	0.122
		Midazolam	106.2	8.3		
SpO2	Before treatment	Dexmedetomidine	98.22	2.32	0.985	0.263
		Midazolam	97.73	3.86		
	During treatment	Dexmedetomidine	97.96	1.97	1.032	0.722
		Midazolam	95.32	3.89		
	After treatment	Dexmedetomidine	99.11	2.90	1.572	0.484
		Midazolam	96.75	4.21		

## Data Availability

The data used in this study are available on request from the corresponding author.

## References

[1] Roizen N. J., Patterson D. (2003). Down’s syndrome. *The Lancet*.

[2] Vicari S. (2006). Motor development and neuropsychological patterns in persons with Down syndrome. *Behavior Genetics*.

[3] Klaiman P., Arndt E. (1989). Facial reconstruction in down syndrome: perceptions of the results by parents and normal adolescents. *Cleft Palate Journal*.

[5] Townsend J. A., Wells M. H. (2019). Behavior guidance of the pediatric dental patient. *Pediatric Dentistry*.

[6] Li B. L., Ni J., Huang J. X., Zhang N., Song X. R., Yuen V. M. (2015). Intranasal dexmedetomidine for sedation in children undergoing transthoracic echocardiography study—a prospective observational study. *Pediatric Anesthesia*.

[7] Coursin D. B., Coursin D. B., Maccioli G. A. (2001). Dexmedetomidine. *Current Opinion in Critical Care*.

[8] Shukry M., Miller J. A. (2010). Update on dexmedetomidine: use in nonintubated patients requiring sedation for surgical procedures. *Therapeutics and Clinical Risk Management*.

[9] Ebert T. J., Hall J., Barney J., Uhrich T., Colinco M. (2000). The effects of increasing plasma concentrations of dexmedetomidine in humans. *Anesthesiology*.

[10] Sheta S. A., Al-Sarheed M. A., Abdelhalim A. A. (2014). Intranasal dexmedetomidine vs. midazolam for premedication in children undergoing complete dental rehabilitation: a double-blinded randomized controlled trial. *Pediatric Anesthesia*.

[11] Natarajan Surendar M., Kumar Pandey R., Kumar Saksena A., Kumar R., Chandra G. (2014). A comparative evaluation of intrnasal dexmedetomidine, midazolam and ketamine for their sedative and analgesic properties: a triple blind randomized study. *Journal of Clinical Pediatric Dentistry*.

[12] Thikkurissy S., Gosnell E. S. (2019). Pain reaction control: sedation. *Pediatric Dentistry*.

[13] Post D. M., Stone L. C., Knutson D. J., Gutierrez T. L., Sari F., Hudson W. A. (2008). Enhancing behavioral science education at the Ohio state university college of medicine. *Academic Medicine*.

[14] Malviya S., Voepel-Lewis T., Tait A., Merkel S., Tremper K., Naughton N. (2002). Depth of sedation in children undergoing computed tomography: validity and reliability of the University of Michigan sedation scale (UMSS). *British Journal of Anaesthesia*.

[15] Miller J., Ding L., Spaeth J. (2017). Sedation methods for transthoracic echocardiography in children with *T* risomy 21—a retrospective study. *Pediatric Anesthesia*.

[16] Mahdavi A., Fallahinejad Ghajari M., Ansari G., Shafiei L. (2018). Intranasal premedication effect of dexmedetomidine versus midazolam on the behavior of 2–6-year-old uncooperative children in dental clinic. *Journal of Dentistry*.

[17] Akin A., Bayram A., Esmaoglu A. (2012). Dexmedetomidine vs. midazolam for premedication of pediatric patients undergoing anesthesia. *Pediatric Anesthesia*.

